# Risankizumab efficacy and safety based on prior inadequate response or intolerance to advanced therapy: post hoc analysis of the INSPIRE and COMMAND phase 3 studies

**DOI:** 10.1093/ecco-jcc/jjaf005

**Published:** 2025-01-13

**Authors:** Remo Panaccione, Edouard Louis, Jean-Frederic Colombel, Geert D’Haens, Laurent Peyrin-Biroulet, Marla Dubinsky, Ken Takeuchi, David T Rubin, Jasmina Kalabic, Karen B Chien, Su Chen, Ling Cheng, Yafei Zhang, W Rachel Duan, Ramona Vladea, Patrick M Hecht, Pierre Morisset, Stefan Schreiber, Marc Ferrante

**Affiliations:** Inflammatory Bowel Disease Unit, Division of Gastroenterology and Hepatology, University of Calgary, Calgary, Alberta, Canada; Hepato-Gastroenterology and Digestive Oncology Department, University Hospital CHU of Liège, Liège, Belgium; Henry D. Janowitz Division of Gastroenterology, Department of Medicine, Icahn School of Medicine at Mount Sinai, New York, NY, United States; Department of Gastroenterology, Amsterdam University Medical Centres, Amsterdam, The Netherlands; Department of Gastroenterology, INFINY Institute, INSERM NGERE, CHRU Nancy, Vandœuvre-lès-Nancy, France; Groupe Hospitalier privé Ambroise Paré - Hartmann, Paris IBD Center, Neuilly sur Seine, France; Division of Gastroenterology and Hepatology, McGill University Health Centre, Montreal, Quebec, Canada; Department of Pediatrics, Inflammatory Bowel Disease Center, Icahn School of Medicine at Mount Sinai, New York, NY, United States; Department of Gastroenterology and Hepatology, IBD Center, Tsujinaka Hospital Kashiwanoha, Kashiwa, Chiba, Japan; University of Chicago Medicine Inflammatory Bowel Disease Center, Chicago, IL, United States; AbbVie Deutschland GmbH & Co. KG, Ludwigshafen, Germany; AbbVie Inc, North Chicago, IL, United States; AbbVie Inc, North Chicago, IL, United States; AbbVie Inc, North Chicago, IL, United States; AbbVie Inc, North Chicago, IL, United States; AbbVie Inc, North Chicago, IL, United States; AbbVie Inc, North Chicago, IL, United States; AbbVie Inc, North Chicago, IL, United States; AbbVie Inc, North Chicago, IL, United States; Department of Internal Medicine, University Hospital Schleswig-Holstein, Kiel University, Kiel, Germany; Department of Gastroenterology and Hepatology, University Hospitals Leuven, KU Leuven, Leuven, Belgium

**Keywords:** Risankizumab, ulcerative colitis, clinical trials

## Abstract

**Background and Aims:**

Treating ulcerative colitis (UC) in patients with prior advanced therapy (AT) exposure may be challenging. We report the efficacy and safety of risankizumab, a monoclonal interleukin 23p19 antibody, in patients with UC and prior inadequate response or intolerance to AT (AT-IR).

**Methods:**

In the 12-week phase 3 INSPIRE induction study, patients were randomized to intravenous risankizumab 1200 mg or placebo. Clinical responders were randomized to subcutaneous risankizumab 180 mg, risankizumab 360 mg, or placebo (risankizumab withdrawal) in the 52-week phase 3 COMMAND maintenance study. This post hoc analysis assessed outcomes by AT-IR status, number, and mechanism of action. AT included biologics, Janus kinase inhibitors, and sphingosine-1-phosphate receptor modulators.

**Results:**

Efficacy analyses included 472 non-AT-IR and 503 AT-IR patients (induction), and 137 non-AT-IR and 411 AT-IR patients (maintenance). More patients achieved clinical remission per Adapted Mayo score with risankizumab 1200 mg versus placebo at induction week 12 (non-AT-IR, 29.7% versus 8.4%, nominal *P* < .0001; AT-IR, 11.4% versus 4.3%, nominal *P* = .0083); consistent with risankizumab 180 mg or risankizumab 360 mg versus placebo (withdrawal) at maintenance week 52 (non-AT-IR, 50.9% or 61.7% versus 31.1%, nominal *P* = .057 or *P* = .0033, respectively; AT-IR, 36.6% or 29.5% versus 23.2%, nominal *P* = .0159 or *P* = .2334, respectively). Risankizumab had increased efficacy over placebo, regardless of AT-IR number or mechanism of action, with higher efficacy rates for non-AT-IR compared to AT-IR. Safety results in non-AT-IR and AT-IR patients were generally comparable in both induction and maintenance.

**Conclusions:**

Risankizumab was effective and well tolerated, regardless of prior AT-IR status.

**Clinical trial registration numbers:**

INSPIRE [NCT03398148], COMMAND [NCT03398135].

## Graphical Abstract

**Graphical Abstract:**
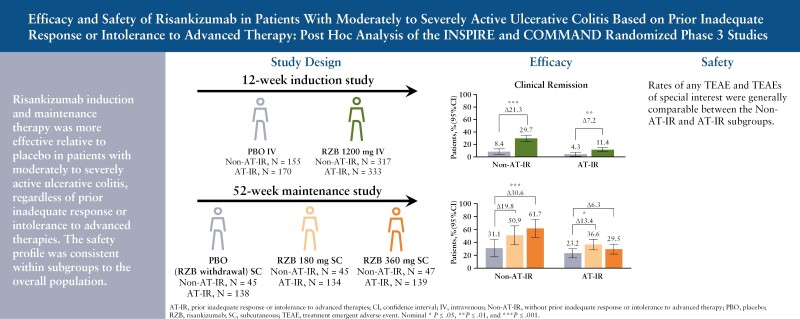

## 1. Introduction

Ulcerative colitis (UC) is a chronic, immune-mediated, relapsing, and remitting inflammatory bowel disease with burdensome symptoms including rectal bleeding, diarrhea, and bowel urgency.^1^ Uncontrolled UC symptoms are associated with quality-of-life impairments.^2^ In 2023, UC was estimated to affect 0.4% of the North American population, with an annual incidence of 15 cases per every 100 000 people.^1^ Conventional UC treatments include aminosalicylates, corticosteroids, and immunomodulators.^3,4^ Advanced therapy (AT) options for UC have been introduced in the past 2 decades; these include antitumor necrosis factor (TNF) therapies, Janus kinase (JAK) inhibitors, anti-integrin therapies, anti-interleukin (IL)-23 and IL-12/23 therapies, and sphingosine-1-phosphate receptor modulators.^4–6^

Despite advances in treatment options for UC, achieving and maintaining disease control is challenging, and patients are often exposed to multiple lines of therapy. It is estimated that 20%–60% of patients with UC achieve an initial clinical response with conventional or AT, representing an apparent therapeutic ceiling.^3,7^ Within 1 year of initiating an AT for UC, approximately 45% of patients will have experienced an inadequate response;^8^ within 2 years, approximately 70% of patients will have received a second-line therapy.^9^

UC is often more difficult to treat in patients with prior exposure to AT.^7^ Biologic monotherapies are often recommended as the first-line AT for patients with UC who are not able to achieve disease control with conventional therapies.^10^ Patients who have had inadequate response to prior AT or biologic therapies experience lower efficacy with subsequent therapies, including anti-integrin therapies (vedolizumab), JAK inhibitors (tofacitinib), anti-IL-12/23 therapies (ustekinumab), and sphingosine-1-phosphate modulators (ozanimod, etrasimod).^11–15^ Additionally, UC may be more refractory to subsequent treatment among patients with multiple prior exposures to ATs.^13,14^ Thus, there is an unmet need for additional therapies that are effective for treating UC among patients with inadequate response or intolerance to prior ATs.

Risankizumab, a humanized IgG1 monoclonal antibody that targets the p19 subunit of IL-23, has been approved for the treatment of Crohn’s disease and UC.^16–21^ Risankizumab demonstrated superior efficacy and consistent safety compared with placebo as induction and maintenance therapy for adults with moderately to severely active UC in the global phase 3 INSPIRE and COMMAND studies.^21^ In this post hoc analysis, we evaluate the efficacy and safety of risankizumab induction and maintenance therapy for patients with UC and prior inadequate response or intolerance to advanced therapy (AT-IR).

## 2. Methods

### 2.1. Study design and treatment

We analyzed data from the phase 3, multicenter, randomized, double-blind, placebo-controlled clinical studies evaluating the efficacy and safety of risankizumab induction and maintenance therapy for adults with moderately to severely active UC. The full trial details, including methods, were previously reported.^21^

In the 12-week phase 3 INSPIRE induction study, patients were randomized 2:1 to receive intravenous (IV) risankizumab 1200 mg or placebo at weeks 0, 4, and 8. Randomization stratification was based on baseline corticosteroid use (yes, no), baseline Adapted Mayo score (≤ 7, > 7), and the number of prior failed ATs (0, 1, > 1). Patients without clinical response per Adapted Mayo score could receive an additional 12 weeks of risankizumab. Patients in the induction study who achieved clinical response after 12 or 24 weeks of risankizumab IV were randomized 1:1:1 to receive subcutaneous (SC) risankizumab 180 mg, risankizumab 360 mg, or placebo every 8 weeks in the 52-week phase 3 COMMAND maintenance study. Randomization stratification was based on the history of AT failure (yes, no), last risankizumab induction dose, and clinical remission status (per local evaluation) at the last visit of induction (yes, no). The induction study consisted of the phase 3 induction reported herein and a preceding phase 2b study, which included a dose-ranging study followed by an open-label substudy; phase 2b study methods and results were previously reported.^21^ Patients, investigators, and study personnel were blinded to the treatment.

The Independent Ethics Committee or Institutional Review Board at each study site approved the study protocol, informed consent forms, and recruitment materials before patient enrollment. The studies were conducted in accordance with the International Conference for Harmonisation guidelines, applicable regulations, and the Declaration of Helsinki. All patients provided written informed consent before screening.

### 2.2. Patients

Eligible patients were aged 18–80 years of age with UC (Adapted Mayo score of 5–9, endoscopic subscore of 2–3) and demonstrated inadequate response or intolerance to aminosalicylates, oral locally acting steroids, systemic steroids, immunomodulators, biologics, or tofacitinib. Exclusion criteria described previously^21^ included prior exposure to p40 inhibitors or p19 inhibitors. The full eligibility criteria were published previously.^21^ There were no restrictions on the number of prior therapies patients could have been exposed to. Patients were categorized by their prior experience with inadequate responses or intolerances to AT, including the presence versus absence of inadequate response or intolerance (AT-IR versus non-AT-IR), the number of prior ATs (1, ≥ 2), the prior AT mechanism of action (TNF inhibitor, vedolizumab, JAK inhibitor), and the specific prior TNF inhibitor therapy (infliximab, adalimumab, or golimumab). ATs included biologics (infliximab, adalimumab, golimumab, vedolizumab), JAK inhibitors (tofacitinib, filgotinib, upadacitinib), and a sphingosine-1-phosphate modulator (ozanimod). Subgroups categorized by AT mechanism of action were not mutually exclusive. Patients who have failed an AT due to lack of efficacy were also assessed.

### 2.3. Assessments

#### 2.3.1. Efficacy assessment

Clinical remission per Adapted Mayo score (stool frequency subscore ≤ 1 and not greater than baseline, rectal bleeding subscore [RBS] of 0, and endoscopic subscore ≤ 1 without evidence of friability), clinical response per Adapted Mayo score (decrease in Adapted Mayo score ≥ 2 points and ≥ 30% from baseline, plus a decrease in RBS ≥ 1 or an absolute RBS ≤ 1), endoscopic improvement (endoscopic subscore of 0 or 1 without evidence of friability), endoscopic remission (endoscopic subscore of 0), and histologic endoscopic mucosal improvement (HEMI; endoscopic subscore of 0 or 1 without the evidence of friability and Geboes score ≤ 3.1) were assessed at week 12 of the induction study and week 52 of the maintenance study.

#### 2.3.2. Safety assessment

Treatment-emergent adverse events (TEAEs), including TEAEs of special interest, and deaths were reported and analyzed for the induction and maintenance studies. TEAEs were adverse events that began on or after the first dose of study drug and within 140 days after the last dose of study drug, or until the first dose of study drug in a subsequent treatment period or study. Adverse events were coded using the Medical Dictionary for Regulatory Activities version 25.1 and summarized using preferred terms.

### 2.4. Statistical analysis

#### 2.4.1. Efficacy analysis

The phase 3 induction efficacy analysis included randomized patients who received ≥ 1 dose of study drug. The maintenance efficacy analysis included randomized patients who achieved clinical response after 12 weeks of risankizumab IV treatment in the phase 2b/3 induction studies and received ≥ 1 dose of study drug in the maintenance study. The number and percentage of patients achieving efficacy endpoints were analyzed for subgroups stratified by AT-IR status. Comparisons between groups were made using Pearson’s chi-square test.

No control for the type I error rate was applied to the exploratory outcomes or the subgroup analyses. Analyses used nonresponder imputation incorporating multiple imputation to handle missing data due to COVID-19 or the geo-political conflict in Ukraine and surrounding impacted regions.

#### 2.4.2. Safety analysis

For the induction study, safety was analyzed in all patients who received ≥ 1 dose of study drug; safety was reported as the number and percentage of TEAEs. For the maintenance study, while the efficacy analysis included all randomized patients who received ≥ 1 dose of study drug during the maintenance study and responded to risankizumab after 12 weeks of IV treatment, safety was analyzed in all randomized patients who received ≥ 1 dose of study drug in the maintenance study after achieving a clinical response following either 12 or 24 weeks of risankizumab IV in the phase 2b or phase 3 induction studies; safety was reported as the number and percentage of TEAEs, and the exposure-adjusted event rates, defined as events per 100 patient-years. Safety outcomes were reported by AT-IR status, and among patients with prior inadequate response or intolerance to JAK inhibitors. Safety data were descriptively summarized with no missing data imputation.

## 3. Results

### 3.1. Patients

The phase 3 induction study enrolled 975 patients who were randomized and received study drug; 650 patients received risankizumab 1200 mg, and 325 patients received placebo (Figure S1). In total, 548 patients with clinical response to 12 weeks of risankizumab IV induction therapy were allocated in the maintenance study to receive risankizumab 180 mg (*N* = 179), risankizumab 360 mg (*N* = 186), or placebo SC (withdrawal; *N* = 183).

In the induction study, the non-AT-IR subgroup included 472 patients (48.4%) and the AT-IR subgroup included 503 patients (51.6%; Table 1); proportions of patients in the AT-IR subgroup were similar between treatment groups (placebo, 52.3%; risankizumab 1200 mg, 51.2%). In the maintenance study, the non-AT-IR subgroup included 137 patients (25.0%) and the AT-IR subgroup included 411 patients (75.0%; Table 2); proportions of patients in the AT-IR subgroup were similar between treatment arms (placebo [withdrawal], 75.4%; risankizumab 180 mg, 74.9%; risankizumab 360 mg, 74.7%). Relative to the non-AT-IR subgroups, patients in the AT-IR subgroups had a longer disease duration, greater prevalence of extensive colitis, higher levels of fecal calprotectin and high-sensitivity C-reactive protein, greater corticosteroid use, and less aminosalicylate use (Tables 1 and 2). Rates of clinical remission at week 0 of maintenance were lower in the AT-IR subgroup compared to the non-AT-IR subgroup (21.1% versus 37.5%); rates of clinical remission at week 0 were also lower in the risankizumab 360 mg group compared to the placebo (withdrawal) group regardless of AT-IR status.

**Table 1.** Baseline patient demographics and disease characteristics for the induction study.

Characteristic	Non-AT-IR, *N* = 472		AT-IR, *N* = 503	
	PBO IV, *N* = 155	RZB 1200 mg IV, *N* = 317	PBO IV, *N* = 170	RZB 1200 mg IV, *N* = 333
Sex, *n* (%)				
Female	54 (34.8)	125 (39.4)	70 (41.2)	140 (42.0)
Male	101 (65.2)	192 (60.6)	100 (58.8)	193 (58.0)
Race, *n* (%)				
American Indian or Alaska Native	0	1 (0.3)	0	0
Asian	65 (41.9)	116 (36.6)	31 (18.2)	55 (16.6)
Black or African American	4 (2.6)	7 (2.2)	3 (1.8)	5 (1.5)
Missing	0	0	0	2
Multiple	3 (1.9)	5 (1.6)	1 (0.6)	0
Native Hawaiian or Pacific Islander	0	0	0	0
White	83 (53.5)	188 (59.3)	135 (79.4)	271 (81.9)
Age, years, median (range)	43.0 (20, 75)	41.0 (18, 77)	41.0 (18, 75)	39.0 (18, 75)
Weight, kg, mean (SD)	72.0 (15.6)	70.5 (16.8)	73.3 (16.5)	73.5 (18.1)
Disease duration, years, median (range)	6.0 (0.3, 37.5)	4.2 (0.0, 42.6)	6.5 (0.6, 32.1)	6.9 (0.4, 39.3)
Tobacco, *n* (%)				
Current	14 (9.3)	36 (11.5)	8 (4.7)	27 (8.2)
Former	35 (23.2)	73 (23.4)	50 (29.4)	83 (25.3)
Never	102 (67.5)	203 (65.1)	112 (65.9)	218 (66.5)
Unknown	4	5	0	5
Disease extent, *n* (%)				
Left-sided	82 (52.9)	170 (53.6)	68 (40.0)	143 (42.9)
Extensive colitis	72 (46.5)	144 (45.4)	102 (60.0)	190 (57.1)
Limited to rectum	1 (0.6)	3 (0.9)	0	0
Fecal calprotectin, mg/kg, median (range)	*N* = 150; 1457.5 (30, 28 800)	*N* = 295; 1369.0 (30, 28 800)	*N* = 152; 1873.5 (44, 28 800)	*N = *307; 1747.0 (30, 28 800)
High-sensitivity C-reactive protein, mg/L, median (range)	*N* = 151; 2.9 (0.2, 107.0)	*N* = 312; 2.7 (0.2, 165.0)	*N* = 167; 5.3 (0.2, 113.0)	*N* = 326; 4.0 (0.2, 199.0)
Adapted Mayo score, *n* (%)				
≤ 7	95 (61.3)	189 (59.6)	95 (55.9)	187 (56.3)
> 7	60 (38.7)	128 (40.4)	75 (44.1)	145 (43.7)
* *Missing	0	0	0	1
Adapted Mayo score, mean (SD)	*N* = 155; 6.9 (1.3)	*N* = 317; 7.0 (1.2)	*N* = 170; 7.2 (1.2)	*N* = 332; 7.2 (1.2)
Endoscopic subscore, *n* (%)				
2	57 (36.8)	134 (42.3)	37 (21.8)	74 (22.2)
3	98 (63.2)	183 (57.7)	133 (78.2)	259 (77.8)
Endoscopic subscore, mean (SD)	2.6 (0.5)	2.6 (0.5)	2.8 (0.4)	2.8 (0.4)
Baseline aminosalicylate use, *n* (%)	139 (89.7)	274 (86.4)	99 (58.2)	201 (60.4)
Baseline immunosuppressant use, *n* (%)	25 (16.1)	61 (19.2)	28 (16.5)	47 (14.1)
Baseline corticosteroid use, *n* (%)	43 (27.7)	99 (31.2)	69 (40.6)	137 (41.1)
Previous advanced therapy inadequate response or intolerance, *n* (%)				
0	155 (100.0)	317 (100.0)	0	0
1	0	0	80 (47.1)	153 (45.9)
2	0	0	55 (32.4)	112 (33.6)
> 2	0	0	35 (20.6)	68 (20.4)
Previous anti-TNF therapy, *n* (%)	6 (3.9)	18 (5.7)	144 (84.7)	287 (86.2)
Adalimumab	2 (1.3)	10 (3.2)	62 (36.5)	115 (34.5)
Golimumab	1 (0.6)	0	22 (12.9)	47 (14.1)
Infliximab	4 (2.6)	9 (2.8)	103 (60.6)	219 (65.8)
Previous JAK inhibitor therapy, *n* (%)	1 (0.6)	3 (0.9)	38 (22.4)	58 (17.4)
Filgotinib	0	0	3 (1.8)	2 (0.6)
Tofacitinib	1 (0.6)	3 (0.9)	33 (19.4)	51 (15.3)
Upadacitinib	0	0	3 (1.8)	5 (1.5)
Previous sphingosine-1-phosphate receptor modulator therapy, *n* (%)	0	0	2 (1.2)	1 (0.3)
* *Ozanimod	0	0	2 (1.2)	1 (0.3)
* *Etrasimod	0	0	0	0
Anti-integrin therapy, *n* (%) Vedolizumab	3 (1.9) 3 (1.9)	5 (1.6) 5 (1.6)	94 (55.3) 94 (55.3)	191 (57.4) 191 (57.4)

AT-IR, with prior inadequate response or intolerance to advanced therapy; IV, intravenous; JAK, Janus kinase; Non-AT-IR, without prior inadequate response or intolerance to advanced therapy; PBO, placebo; RZB, risankizumab; TNF, tumor necrosis factor; *N* = number of patients assessed.

Includes all randomized patients who received ≥ 1 dose of the study drug.

**Table 2.** Baseline patient demographics and disease characteristics for the maintenance study.

Characteristic	Non-AT-IR *N* = 137			AT-IR *N* = 411		
	PBO (Withdrawal) SC *n* = 45	RZB 180 mg SC *n* = 45	RZB 360 mg SC *n* = 47	PBO (Withdrawal) SC *n* = 138	RZB 180 mg SC *n* = 134	RZB 360 mg SC *n* = 139
Sex, n (%)						
Female	18 (40.0)	15 (33.3)	19 (40.4)	64 (46.4)	59 (44.0)	60 (43.2)
Male	27 (60.0)	30 (66.7)	28 (59.6)	74 (53.6)	75 (56.0)	79 (56.8)
Race, n (%)						
American Indian or Alaska Native	0	0	0	0	0	0
Asian	19 (42.2)	18 (40.0)	27 (57.4)	27 (19.6)	18 (13.4)	24 (17.3)
Black or African American	0	1 (2.2)	0	2 (1.4)	3 (2.2)	1 (0.7)
Multiple	0	0	1 (2.1)	0	0	0
Native Hawaiian or Pacific Islander	0	0	0	0	0	0
White	26 (57.8)	26 (57.8)	19 (40.4)	109 (79.0)	113 (84.3)	114 (82.0)
Age, years, median (range)	39.0 (21, 68)	41.0 (23, 64)	44.0 (20, 73)	36.0 (18, 79)	38.0 (18, 79)	40.0 (19, 78)
Weight, kg, mean (SD)	70.0 (16.3)	73.5 (18.5)	67.4 (18.4)	69.7 (17.2)	71.9 (16.8)	72.4 (16.7)
Disease duration, years, median (range)	5.7 (0.4, 42.6)	3.7 (0.3, 25.8)	4.4 (0.6, 26.0)	6.4 (0.6, 35.2)	7.9 (0.8, 52.5)	7.8 (0.8, 34.2)
Tobacco, *n* (%)						
Current	32 (71.1)	31 (70.5)	25 (54.3)	91 (66.9)	96 (72.2)	87 (64.4)
Former	3 (6.7)	3 (6.8)	7 (15.2)	13 (9.6)	7 (5.3)	11 (8.1)
Never	10 (22.2)	10 (22.7)	14 (30.4)	32 (23.5)	30 (22.6)	37 (27.4)
Unknown	0	1	1	2	1	4
Disease extent, *n* (%)						
Left-sided	30 (66.7)	28 (62.2)	27 (57.4)	55 (39.9)	56 (41.8)	65 (46.8)
Extensive colitis	15 (33.3)	17 (37.8)	20 (42.6)	83 (60.1)	77 (57.5)	74 (53.2)
Limited to rectum	0	0	0	0	1 (0.7)	0
Fecal calprotectin, mg/kg, median (range)	*N* = 44; 1492.5 (30, 14 314)	*N* = 41; 1585.0 (30, 9 073)	*N* = 43; 1111.0 (30, 28 800)	*N* = 118; 1518.5 (30, 28 800)	*N* = 110; 1621.5 (30, 28 800)	*N* = 123; 1771.0 (30, 28 800)
High-sensitivity C-reactive protein, mg/L, median (range)	*N* = 43; 2.2 (0.2, 40.8)	*N* = 45; 2.6 (0.2, 82.2)	*N* = 47; 1.3 (0.2, 39.4)	*N* = 136; 6.2 (0.2, 167.0)	*N* = 132; 5.3 (0.2, 119.0)	*N* = 137; 3.8 (0.2, 113.0)
Clinical remission at week 0 of maintenance, *n* (%)	18 (40.0)	18 (40.9)	15 (31.9)	35 (25.7)	26 (19.4)	25 (18.1)
Adapted Mayo Score, *n* (%)						
≤ 7	25 (55.6)	33 (73.3)	26 (55.3)	67 (48.6)	69 (51.5)	83 (59.7)
> 7	20 (44.4)	12 (26.7)	21 (44.7)	71 (51.4)	65 (48.5)	56 (40.3)
Adapted Mayo score, mean (SD)	7.0 (1.3)	6.8 (1.1)	7.0 (1.3)	7.3 (1.2)	7.3 (1.2)	7.0 (1.2)
Endoscopic subscore, *n* (%)						
2	16 (35.6)	18 (40.0)	19 (40.4)	36 (26.1)	29 (21.6)	44 (31.7)
3	29 (64.4)	27 (60.0)	28 (59.6)	102 (73.9)	105 (78.4)	95 (68.3)
Endoscopic subscore, mean (SD)	2.6 (0.5)	2.6 (0.5)	2.6 (0.5)	2.7 (0.4)	2.8 (0.4)	2.7 (0.5)
Baseline aminosalicylate use, *n* (%)	42 (93.3)	38 (84.4)	43 (91.5)	75 (54.3)	81 (60.4)	92 (66.2)
Baseline immunosuppressant use, *n* (%)	9 (20.0)	9 (20.0)	4 (8.5)	28 (20.3)	26 (19.4)	28 (20.1)
Baseline corticosteroid use, *n* (%)	9 (20.0)	15 (33.3)	9 (19.1)	59 (42.8)	59 (44.0)	50 (36.0)
Previous advanced therapy inadequate response or intolerance, *n* (%)						
0	45 (100.0)	45 (100.0)	47 (100.0)	0	0	0
1	0	0	0	62 (44.9)	52 (38.8)	55 (39.6)
2	0	0	0	40 (29.0)	44 (32.8)	37 (26.6)
> 2	0	0	0	36 (26.1)	38 (28.4)	47 (33.8)
Previous anti-TNF therapy, *n* (%)	8 (17.8)	4 (8.9)	4 (8.5)	131 (94.9)	124 (92.5)	129 (92.8)
Adalimumab	3 (6.7)	2 (4.4)	2 (4.3)	63 (45.7)	70 (52.2)	59 (42.4)
Golimumab	2 (4.4)	0	1 (2.1)	26 (18.8)	21 (15.7)	19 (13.7)
Infliximab	6 (13.3)	2 (4.4)	3 (6.4)	97 (70.3)	88 (65.7)	102 (73.4)
Previous JAK inhibitor therapy, *n* (%)	0	0	1 (2.1)	31 (22.5)	23 (17.2)	28 (20.1)
Filgotinib	0	0	0	1 (0.7)	0	1 (0.7)
Tofacitinib	0	0	1 (2.1)	29 (21.0)	22 (16.4)	26 (18.7)
Upadacitinib	0	0	0	1 (0.7)	1 (0.7)	1 (0.7)
Previous sphingosine-1-phosphate receptor modulator therapy, *n* (%)	0	0	0	1 (0.7)	1 (0.7)	3 (2.2)
Ozanimod	0	0	0	1 (0.7)	1 (0.7)	3 (2.2)
Etrasimod	0	0	0	0	0	0
Anti-integrin therapy, *n* (%)	1 (2.2)	1 (2.2)	3 (6.4)	69 (50.0)	67 (50.0)	81 (58.3)
Vedolizumab	1 (2.2)	1 (2.2)	3 (6.4)	69 (50.0)	67 (50.0)	81 (58.3)

AT-IR, with prior inadequate response or intolerance to advanced therapy; Non-AT-IR, without prior inadequate response or intolerance to advanced therapy; PBO, placebo; RZB, risankizumab; SC, subcutaneous; TNF, tumor necrosis factor; *N* = number of patients assessed.

Includes all randomized patients who received ≥ 1 dose of the study drug.

Among patients who did not meet the criteria for experiencing AT-IR, the prevalence of prior AT exposure was 6.8% in the induction study (placebo, 5.2%; risankizumab 1200 mg, 7.6%) and 13.1% in the maintenance study (placebo [withdrawal], 17.8%; risankizumab 180 mg, 8.9%; risankizumab 360 mg, 12.8%). Among the induction study AT-IR subgroup, 46.3%, 33.2%, and 20.5% of patients experienced AT-IR with 1, 2, and > 2 therapies, respectively; proportions were similar between treatment groups (Table 1). Among the maintenance study AT-IR subgroup, 41.1%, 29.4%, and 29.4% of patients experienced AT-IR with 1, 2, and > 2 therapies, respectively; the proportion of patients who experienced AT-IR with > 2 therapies was higher in the risankizumab 360 mg group compared to the other treatment groups (Table 2). In the induction study AT-IR subgroup, 85.7% of patients had prior exposure to anti-TNF therapy, 56.7% had prior exposure to vedolizumab, and 19.1% had prior exposure to JAK inhibitors (Table 1). In the maintenance study AT-IR subgroup, 93.4% of patients had prior exposure to anti-TNF therapy, 52.8% had prior exposure to vedolizumab, and 20.0% had prior exposure to JAK inhibitors (Table 2). Missing data for these patients are detailed in Table S1.

### 3.2. Efficacy

#### 3.2.1. Induction efficacy outcomes

**Figure 1.**
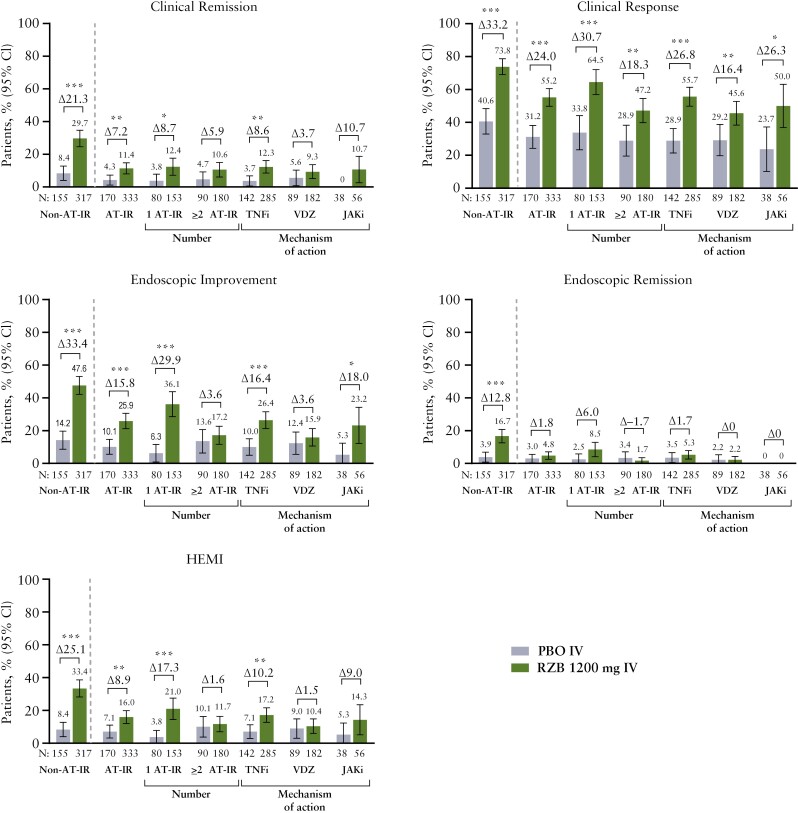
Week 12 efficacy outcomes by patient experience with AT-IR. AT-IR, with prior inadequate response or intolerance to advanced therapy; HEMI, histologic endoscopic mucosal improvement; IV, intravenous; JAKi, Janus kinase inhibitor; Non-AT-IR, without prior inadequate response or intolerance to advanced therapy; PBO, placebo; RZB, risankizumab; TNFi, tumor necrosis factor inhibitor; VDZ, vedolizumab. Nominal *P ≤ .05, **P ≤ .01, and ***P ≤ .001.

At week 12 of induction, clinical remission was achieved by greater proportions of patients treated with risankizumab 1200 mg compared to patients treated with placebo in the non-AT-IR and AT-IR subgroups (non-AT-IR: 29.7% versus 8.4%, nominal *P* < .0001; AT-IR: 11.4% versus 4.3%, nominal *P* = .0083; Figure 1). In the non-AT-IR subgroup, 47.6% of risankizumab-treated patients achieved endoscopic improvement at week 12. Regardless of whether patients had experienced AT-IR, the proportion who achieved clinical response, endoscopic improvement, endoscopic remission, and HEMI was higher in the risankizumab 1200 mg group compared to the placebo group. In the AT-IR subgroup, clinical and endoscopic outcomes were generally achieved by a higher proportion of patients in the risankizumab 1200 mg group compared to the placebo group irrespective of the number of prior AT failures (1, ≥ 2). Similarly, clinical and endoscopic outcomes were achieved with risankizumab 1200 mg across AT-IR subgroups categorized by AT mechanism of action (TNF inhibitor, vedolizumab, JAK inhibitor) or specific TNF inhibitor type (infliximab, adalimumab, golimumab); these subgroups were not mutually exclusive (Figures 1 and S2).

Rates of clinical remission at week 12 with risankizumab 1200 mg were higher in the non-AT-IR subgroup relative to the AT-IR subgroup (29.7% versus 11.4%), and comparable in patients with inadequate response or intolerance to 1 versus ≥ 2 ATs (12.4% versus 10.6%); trends were generally similar for clinical response, endoscopic improvement, endoscopic remission, and HEMI (Figure 1). There were no consistent patterns in efficacy rates based on the prior failed therapy mechanism of action (TNF inhibitor, vedolizumab, JAK inhibitor), or specific TNF inhibitor type (infliximab, adalimumab, golimumab) (Figure S2).

#### 3.2.2. Maintenance efficacy outcomes

**Figure 2.**
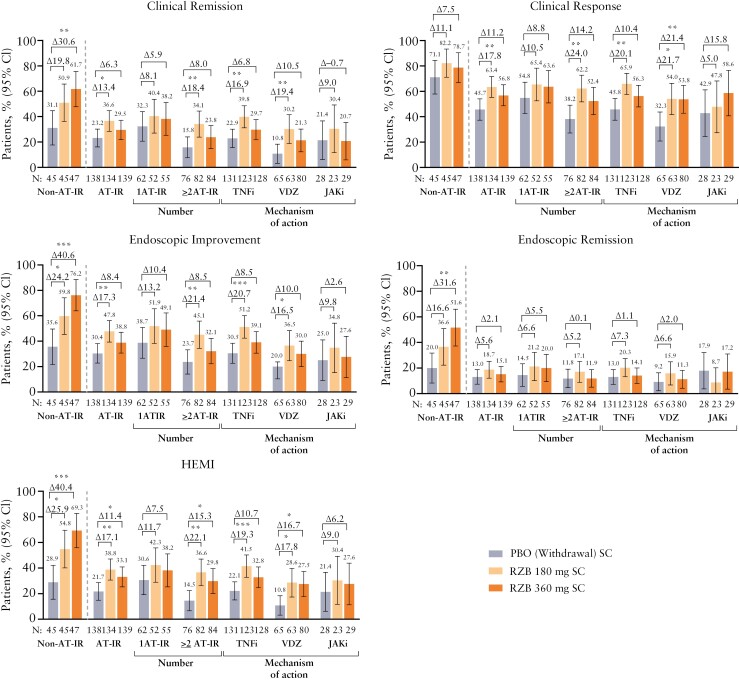
Week 52 efficacy outcomes by patient experience with AT-IR. AT-IR, with prior inadequate response or intolerance to advanced therapy; HEMI, histologic endoscopic mucosal improvement; JAKi, Janus kinase inhibitor; Non-AT-IR, without prior inadequate response or intolerance to advanced therapy; PBO, placebo; RZB, risankizumab; SC, subcutaneous; TNFi, tumor necrosis factor inhibitor; VDZ, vedolizumab. Nominal *P ≤ .05, **P ≤ .01, and ***P ≤ .001.

At week 52 of the maintenance study, rates of achieving clinical remission were higher with risankizumab 180 mg or risankizumab 360 mg versus placebo (withdrawal) in the non-AT-IR subgroup (50.9% or 61.7% versus 31.1%; nominal *P* = .057 or nominal *P* = .0033, respectively) and the AT-IR subgroup (36.6% or 29.5% versus 23.2%; nominal *P* = .0159 or nominal *P* = .2334, respectively; Figure 2). In the non-AT-IR subgroup, 59.8% of patients treated with risankizumab 180 mg and 76.2% of patients treated with risankizumab 360 mg achieved endoscopic improvement at week 52 relative to 35.6% of patients treated with placebo. Among the AT-IR patients, 47.8% of patients treated with risankizumab 180 mg and 38.8% of patients treated with risankizumab 360 mg achieved endoscopic improvement at week 52 relative to 30.4% of patients treated with placebo. Additionally, irrespective of whether patients had experienced AT-IR, the proportion who achieved clinical response, endoscopic remission, and HEMI was higher in the risankizumab 180 mg or risankizumab 360 mg groups versus the placebo (withdrawal) group. In the AT-IR subgroup, rates of achieving clinical and endoscopic outcomes were generally higher with either dose of risankizumab versus placebo (withdrawal), regardless of the prior failure to AT number (1, ≥ 2), mechanism of action (TNF inhibitor, vedolizumab, JAK inhibitor), or specific TNF inhibitor type (infliximab, adalimumab, golimumab; Figures 2 and S3).

At week 52, the proportion of patients achieving clinical and endoscopic outcomes with risankizumab 180 mg or risankizumab 360 mg was higher for the non-AT-IR versus the AT-IR subgroup (Figure 2). In the AT-IR subgroup, there were no clear patterns in the efficacy of risankizumab maintenance therapy based on prior AT number (1, ≥ 2). Similarly, there were no clear patterns across clinical and endoscopic outcomes with risankizumab maintenance therapy across AT-IR subgroups by mechanism of action (TNF inhibitor, vedolizumab, JAK inhibitor) or specific TNF inhibitor type (infliximab, adalimumab, golimumab); these subgroups were not mutually exclusive (Figures 2 and S3).

### 3.3. Safety

#### 3.3.1. Induction safety outcomes

The proportions of patients with any TEAE in the induction study were comparable between the non-AT-IR and AT-IR subgroups in both the risankizumab 1200 mg treatment group (39.9% and 44.1%) and the placebo treatment group (45.5% and 53.5%; Table 3). Rates of TEAEs of special interest were generally similar between the non-AT-IR and AT-IR subgroups (Table 3). One treatment-emergent death due to COVID-19-related respiratory failure occurred in a patient in the AT-IR subgroup who was receiving risankizumab 1200 mg (Table 3). Among the AT-IR subgroup, TEAE rates were comparable between subgroups with 1 AT-IR and ≥ 2 AT-IR (Table S2). The incidence of TEAEs among patients with inadequate response or intolerance to a JAK inhibitor was comparable to the incidence of TEAEs among patients in the overall AT-IR subgroup (Tables S3 and 3).

**Table 3.** Overview of adverse events for the induction study

Patients, *n* (%)	Non-AT-IR		AT-IR	
	PBO IV *N* = 154	RZB 1200 mg IV *N* = 318	PBO IV *N* = 170	RZB 1200 mg IV *N* = 333
Overview of treatment-emergent adverse events				
Any AE	70 (45.5)	127 (39.9)	91 (53.5)	147 (44.1)
Severe AE	13 (8.4)	12 (3.8)	20 (11.8)	4 (1.2)
Serious AE	13 (8.4)	10 (3.1)	20 (11.8)	5 (1.5)
AE possibly related to the study drug as assessed by the investigator	11 (7.1)	20 (6.3)	15 (8.8)	41 (12.3)
AE leading to study drug discontinuation	5 (3.2)	1 (0.3)	7 (4.1)	3 (0.9)
AE related to COVID-19	8 (5.2)	12 (3.8)	11 (6.5)	23 (6.9)
Death	0	0	0	1 (0.3)^a^
Treatment-emergent adverse events of special interest				
Adjudicated MACE	0	0	0	0
Serious infections	2 (1.3)	2 (0.6)	2 (1.2)	2 (0.6)
Active tuberculosis	0	0	0	0
Opportunistic infection^b^	0	0	0	0
Herpes zoster	0	1 (0.3)	0	1 (0.3)
Malignancies	0	0	2 (1.2)	0
NMSC	0	0	0	0
Hypersensitivity	2 (1.3)	7 (2.2)	4 (2.4)	17 (5.1)
Serious hypersensitivity	0	0	0	0
Adjudicated anaphylactic reactions	0	0	0	0
Hepatic events	4 (2.6)	4 (1.3)	10 (5.9)	6 (1.8)
Injection site reactions	2 (1.3)	1 (0.3)	2 (1.2)	3 (0.9)

AE, adverse event; AT-IR, with prior inadequate response or intolerance to advanced therapy; MACE, major adverse cardiovascular event; NMSC, nonmelanoma skin cancer; Non-AT-IR, without prior inadequate response or intolerance to advanced therapy; PBO, placebo; RZB, risankizumab.

Includes all patients who received ≥ 1 dose of the study drug in the induction study.

^a^Death was caused by respiratory failure due to COVID-19.

^b^Excluding tuberculosis and herpes zoster.

#### 3.3.2. Maintenance safety outcomes

In the maintenance study, the proportions of patients with any TEAE were generally comparable between the non-AT-IR and AT-IR subgroups in the risankizumab 180 mg treatment group (66.0% and 74.7%), risankizumab 360 mg treatment group (75.0% and 69.4%), and placebo (withdrawal) treatment group (76.6% and 76.5%; Table 4); exposure-adjusted rates of TEAEs are provided in Table S4. Rates of TEAEs of special interest were generally similar between the non-AT-IR and AT-IR subgroups (Table 4) with the exception of the higher rates of hypersensitivity and hepatic events among patients receiving risankizumab 360 mg in the non-AT-IR subgroup. The majority of hepatic events were isolated elevations in liver chemistry tests and none were assessed as serious. One nontreatment-emergent death due to colon adenocarcinoma (considered unrelated to the study drug) occurred in a patient in the AT-IR risankizumab 360 mg group (Table 4). Rates of any TEAE and TEAEs of special interest were generally consistent between subgroups with 1 and ≥ 2 AT-IRs (Table S5). While the incidence of TEAEs among patients with inadequate response or intolerance to a JAK inhibitor was higher than the incidence of TEAEs among patients in the overall AT-IR subgroup, rates of severe and serious TEAEs and TEAEs leading to discontinuation were comparable between these subgroups (Tables S3 and 4).

**Table 4.** Overview of adverse events for the maintenance study.

Patients, *n* (%)	Non-AT-IR			AT-IR		
	PBO (Withdrawal) SC *N* = 47	RZB 180 mg SC *N* = 47	RZB 360 mg SC *N* = 48	PBO (Withdrawal) SC *N* = 149	RZB 180 mg SC *N* = 146	RZB 360 mg SC *N* = 147
Overview of treatment-emergent adverse events						
Any AE	36 (76.6)	31 (66.0)	36 (75.0)	114 (76.5)	109 (74.7)	102 (69.4)
Severe AE	0	1 (2.1)	0	10 (6.7)	2 (1.4)	6 (4.1)
Serious AE	0	4 (8.5)	0	16 (10.7)	6 (4.1)	10 (6.8)
AE possibly related to the study drug as assessed by the investigator	6 (12.8)	6 (12.8)	9 (18.8)	35 (23.5)	30 (20.5)	25 (17.0)
AE leading to study drug discontinuation	0	0	0	3 (2.0)	3 (2.1)	5 (3.4)
AE related to COVID-19	10 (21.3)	7 (14.9)	10 (20.8)	17 (11.4)	13 (8.9)	16 (10.9)
Death	0	0	0	0	0	1 (0.7)^a^
Treatment-emergent adverse events of special interest						
Adjudicated MACE	0	0	0	0	0	0
Serious infections	0	1 (2.1)	0	4 (2.7)	1 (0.7)	1 (0.7)
Active tuberculosis	0	0	0	0	0	0
Opportunistic infection^b^	0	0	0	0	0	1 (0.7)
Herpes zoster	0	1 (2.1)	0	3 (2.0)	1 (0.7)	1 (0.7)
Malignancies	0	0	0	1 (0.7)	0	2 (1.4)
NMSC	0	0	0	1 (0.7)	0	0
Hypersensitivity	1 (2.1)	1 (2.1)	5 (10.4)	9 (6.0)	19 (13.0)	5 (3.4)
Serious hypersensitivity	0	0	0	0	0	0
Adjudicated anaphylactic reactions	0	0	0	0	0	0
Hepatic events	0	2 (4.3)	7 (14.6)	1 (0.7)	1 (0.7)	6 (4.1)
Injection site reactions	0	1 (2.1)	1 (2.1)	2 (1.3)	6 (4.1)	4 (2.7)

AE, adverse event; AT-IR, with prior inadequate response or intolerance to advanced therapy; MACE, major adverse cardiovascular event; NMSC, nonmelanoma skin cancer; Non-AT-IR, without prior inadequate response or intolerance to advanced therapy; PBO, placebo; RZB, risankizumab.

Includes all randomized patients who received IV RZB in the induction study and who also received ≥ 1 dose of study drug in the maintenance study.

^a^Death was due to adenocarcinoma of the colon, considered unrelated to the study drug.

^b^Excluding tuberculosis and herpes zoster.

When data were presented separately by failure type (defined as patients who have failed an AT due to lack of efficacy) (Figure S4, Table S6), results from this subgroup were similar to the overall AT-IR group (Tables 3 and 4).

## 4. Discussion

Results from this post hoc analysis demonstrate that risankizumab is effective and well tolerated for the treatment of UC in the non-AT-IR and AT-IR subgroups during induction and maintenance. Among patients in the AT-IR subgroup, risankizumab induction and maintenance therapy were generally more effective than placebo at achieving clinical and endoscopic outcomes, irrespective of the prior AT number (1, ≥ 2), mechanism of action (TNF inhibitor, vedolizumab, JAK inhibitor), or specific TNF inhibitor type (infliximab, adalimumab, golimumab). These results indicate that risankizumab was effective for treating UC, even among patients who were refractory to multiple prior ATs. The safety profile of risankizumab was similar in the non-AT-IR and AT-IR subgroups and was consistent with findings from the primary publication.^21^

In this post hoc analysis, results suggested that the proportion of patients achieving clinical remission and clinical response with risankizumab induction and maintenance therapy was higher for the non-AT-IR subgroup compared to the AT-IR subgroup. Endoscopic outcomes were higher for the non-AT-IR subgroup, with nearly half of the patients treated with risankizumab achieving endoscopic improvement at week 12 and up to three-quarters of patients in maintenance achieving endoscopic improvement at week 52. In alignment with other studies, data from this analysis suggests that UC may be more difficult to treat in patients who have experienced intolerance or an inadequate response to prior ATs. The rates of symptomatic remission of UC with the IL-12/23 antagonist ustekinumab were higher in biologic-naïve patients compared with biologic-exposed patients.^11^ During induction, the efficacy rates for the JAK inhibitor upadacitinib were generally higher for patients without a prior inadequate response to AT compared to patients with a prior inadequate response to AT.^22^ Similarly, efficacy rates for tofacitinib, vedolizumab, ozanimod, and etrasimod were higher for patients without prior biologic exposure or failure compared to patients with prior biologic exposure or failure.^12–15^ However, direct comparisons of the results presented herein with those from other clinical trials are limited by differences in trial design and the lack of a head-to-head study.

A greater proportion of patients who received 1 prior AT compared to patients who received ≥ 2 prior ATs achieved clinical and endoscopic outcomes with risankizumab induction therapy compared to patients with less experience with ATs. These findings along with the differences in efficacy observed between the non-AT-IR and AT-IR patients suggest that patients with AT-IR, and especially those with inadequate response or intolerance to multiple therapies, may have more refractory disease in general. This result is corroborated by results that show that the AT-IR subgroups had a longer disease duration, more extensive and severe disease, and higher levels of inflammatory biomarkers relative to patients in the non-AT-IR subgroups. Risankizumab consistently demonstrated a benefit over placebo, regardless of whether patients had previously experienced AT-IR with multiple therapies. However, it is worth mentioning that the differences observed in patients who had prior failure to ≥ 2 ATs were numerically less.

Prior treatment exposure and response may be important effect modifiers for patients with UC.^4,23,24^ Given the expanding number of AT options for UC, it is important to consider the efficacy of risankizumab among patients grouped by the type of prior ATs to which they were exposed.^4,23,24^ To date, the analysis reported here has enrolled most patients with moderately to severely active UC for treatment with risankizumab who had prior exposure to a JAK inhibitor. Regardless of the mechanism of action of prior therapies that patients experienced inadequate response or intolerance to, risankizumab induction and maintenance therapy was generally more effective than placebo for treating UC.

Making informed treatment decisions for patients with UC who have received prior therapies can be challenging; there are limited data evaluating the efficacy of UC therapies based on the number, mechanism of action, or specific type of therapy to which patients were previously exposed. Most reports of the efficacy of UC therapies among patients with prior biologic or AT exposure include fewer than 400 patients.^11–13,15^ While a study of tofacitinib for UC included more than 550 patients categorized by the number of prior TNF inhibitor failures, neither the specific TNF inhibitors nor prior therapies with other mechanisms of action were considered in the primary analysis.^14^ In the phase 3 studies evaluating risankizumab for UC, over 50% of patients in the induction study had experienced AT-IR, and 75% of patients in the maintenance study had experienced AT-IR (a higher proportion than in other phase 3 maintenance studies evaluating UC treatments).^21^ The present analysis describes the outcomes of risankizumab as induction and maintenance therapy in 503 and 411 patients with AT-IR, respectively, which allowed for further stratification based on the number, mechanism of action, and specific AT to which patients experienced inadequate response or intolerance. The comprehensive results herein may help inform treatment decisions for patients who did not respond to their initial therapy for UC.

A limitation of our analysis is that it was conducted post hoc. Additionally, while the non-AT-IR and AT-IR subgroups had large numbers of patients, some subgroups stratified by therapy mechanism of action or specific therapy had limited patient numbers. Subgroups categorized by AT mechanism of action were not mutually exclusive. In the maintenance study, characteristics of patients receiving risankizumab 360 mg suggest these patients may have had more refractory disease compared with patients in other treatment groups, which could have influenced outcomes. For example, relative to patients in other treatment groups, patients receiving risankizumab 360 mg had lower rates of clinical remission at week 0 of maintenance and a higher prevalence of having an inadequate response or intolerance with > 2 ATs. Based on our analysis, it cannot be distinguished whether any observed outcome differences in patients with AT-IR compared to those without AT-IR are due to prior treatment experience or due to other disease characteristics that may be more prevalent among patients with more refractory disease. Additional limitations, in the maintenance trial, were that drug levels of risankizumab were detectable in the placebo group up to week 16, as these patients received risankizumab during the induction trial but not during the maintenance trial.^21^ This ongoing exposure to risankizumab from the induction trial could potentially inflate the response rates in the placebo group during the maintenance trial.^21,25^ Additionally, there was persistent suppression of serum concentrations of IL-22, a cytokine and biomarker for anti-IL-23 therapy, observed from week 4 to week 52.^21^ Maintenance study results, for the post hoc analysis herein as well as the primary report, should be interpreted in the context of the risankizumab pharmacokinetic exposure-response relationship; while efficacy increases with risankizumab exposure, the difference in efficacy between the risankizumab 180 mg and 360 mg doses is modest.^26^

In conclusion, compared to placebo, risankizumab was effective and well tolerated for the treatment of patients with moderately to severely active UC, regardless of prior inadequate response or intolerance to ATs. Increased efficacy in induction and maintenance was observed for risankizumab over placebo for the treatment of UC in patients who experienced inadequate response or intolerance to prior ATs irrespective of the number, mechanism of action, or specific therapy.

## Supplementary Material

jjaf005_suppl_Supplementary_Material

## Data Availability

AbbVie is committed to responsible data sharing regarding the clinical trials we sponsor. This includes access to anonymized individual and trial-level data (analysis data sets), as well as other information (eg, protocols, clinical study reports, or analysis plans), as long as the trials are not part of an ongoing or planned regulatory submission. This includes requests for clinical trial data for unlicensed products and indications. These clinical trial data can be requested by any qualified researchers who engage in rigorous, independent, scientific research, and will be provided following review and approval of a research proposal and Statistical Analysis Plan (SAP) and execution of a Data Sharing Agreement (DSA). Data requests can be submitted at any time after approval in the United States and Europe and after acceptance of this manuscript for publication. The data will be accessible for 12 months, with possible extensions considered. For more information on the process or to submit a request, visit the following link: https://vivli.org/ourmember/abbvie/, then select “Home.”
