# Supplementary material for: Risankizumab efficacy and safety based on prior inadequate response or intolerance to advanced therapy: post hoc analysis of the INSPIRE and COMMAND phase 3 studies
Source: J Crohns Colitis. 2025 Jan 13;19(1):jjaf005. doi: 10.1093/ecco-jcc/jjaf005 (PMC11772864; doi:10.1093/ecco-jcc/jjaf005)
Supplement: jjaf005_suppl_Supplementary_Material [file jjaf005_suppl_supplementary_material.docx]

| **Table S1. Number of Patients With Missing Data for the Primary Endpoint** | | | | | | | | | | | |
| --- | --- | --- | --- | --- | --- | --- | --- | --- | --- | --- | --- |
|  | **Week 12 of Induction** | | | |  | **Week 52 of Maintenance** | | | | | |
| **Endpoint, n (%)** | **Non-AT-IR N = 472** | | **AT-IR**  **N = 503** | |  | **Non-AT-IR N = 137** | | | **AT-IR**  **N = 411** | | |
|  | **PBO**  **IV**  **N = 155** | **RZB 1200 mg IV**  **N = 317** | **PBO**  **IV**  **N = 170** | **RZB 1200 mg IV**  **N = 333** |  | **PBO (Withdrawal) SC**  **N = 45** | **RZB 180 mg SC**  **N = 45** | **RZB 360 mg SC**  **N = 47** | **PBO (Withdrawal) SC**  **N = 138** | **RZB 180 mg SC**  **N = 134** | **RZB 360 mg SC**  **N = 139** |
| Clinical remission per Adapted Mayo score | 6 (3.9) | 10 (3.2) | 12 (7.1) | 5 (1.5) |  | 4 (8.9) | 6 (13.3) | 2 (4.3) | 19 (13.8) | 12 (9.0) | 23 (16.5) |
| AT-IR, with prior inadequate response or intolerance to advanced therapy; IV, intravenous; Non-AT-IR, without prior inadequate response or intolerance to advanced therapy; PBO, placebo; RZB, risankizumab; SC, subcutaneous.  Includes all randomized patients who received ≥ 1 dose of the study drug.  Data are presented as number of patients who achieved clinical remission per Adapted Mayo score. | | | | | | | | | | | |

| **Table S2. Overview of Adverse Events Based on Number of Prior Advanced Therapies for the Induction Study** | | | | |
| --- | --- | --- | --- | --- |
| **Patients, n (%)** | **AT-IR = 1** | | **AT-IR ≥ 2** | |
|  | **PBO IV**  **N = 80** | **RZB**  **1200 mg IV**  **N = 153** | **PBO IV**  **N = 90** | **RZB**  **1200 mg IV**  **N = 180** |
| Overview of treatment-emergent adverse events | | | | |
| *Any AE* | 41 (51.3) | 63 (41.2) | 50 (55.6) | 84 (46.7) |
| *Severe AE* | 6 (7.5) | 2 (1.3) | 14 (15.6) | 2 (1.1) |
| *Serious AE* | 7 (8.8) | 1 (0.7) | 13 (14.4) | 4 (2.2) |
| *AE possibly related to the study drug as assessed by the investigator* | 7 (8.8) | 15 (9.8) | 8 (8.9) | 26 (14.4) |
| *AE leading to study drug discontinuation* | 2 (2.5) | 1 (0.7) | 5 (5.6) | 2 (1.1) |
| *AE related to COVID-19* | 6 (7.5) | 9 (5.9) | 5 (5.6) | 14 (7.8) |
| *Death* | 0 | 1 (0.7)^a^ | 0 | 0 |
| Treatment-emergent adverse events of special interest | | | | |
| *Adjudicated MACE* | 0 | 0 | 0 | 0 |
| *Serious infections* | 0 | 1 (0.7) | 2 (2.2) | 1 (0.6) |
| *Active tuberculosis* | 0 | 0 | 0 | 0 |
| *Opportunistic infection^b^* | 0 | 0 | 0 | 0 |
| *Herpes zoster* | 0 | 0 | 0 | 1 (0.6) |
| *Malignancies* | 1 (1.3) | 0 | 1 (1.1) | 0 |
| *NMSC* | 0 | 0 | 0 | 0 |
| *Hypersensitivity* | 3 (3.8) | 7 (4.6) | 1 (1.1) | 10 (5.6) |
| *Serious hypersensitivity* | 0 | 0 | 0 | 0 |
| *Adjudicated anaphylactic reactions* | 0 | 0 | 0 | 0 |
| *Hepatic events* | 3 (3.8) | 2 (1.3) | 7 (7.8) | 4 (2.2) |
| *Injection site reactions* | 2 (2.5) | 2 (1.3) | 0 | 1 (0.6) |

| AE, adverse event; AT-IR, with prior inadequate response or intolerance to advanced therapy; MACE, major adverse cardiovascular event; NMSC, nonmelanoma skin cancer; PBO, placebo; RZB, risankizumab.  Includes all patients who received ≥ 1 dose of the study drug in the induction study.  ^a^Death was caused by respiratory failure due to COVID-19.  ^b^Excluding tuberculosis and herpes zoster. |
| --- |

| **Table S3. Overview of Adverse Events in Patients With Prior Inadequate Response or Intolerance to a JAK Inhibitor** | | | | | | |
| --- | --- | --- | --- | --- | --- | --- |
| **Patients, n (%); E/100 PY** | **Induction Study^a^** | | **Maintenance Study^b^** | | | |
|  | **PBO IV**  **N = 38** | **RZB 1200 mg IV**  **N = 56** | | **PBO (Withdrawal) SC**  **N = 35**  **PY = 29.5** | **RZB 180 mg SC**  **N = 26**  **PY = 22.6** | **RZB 360 mg SC**  **N = 31**  **PY = 26.7** |
| Overview of treatment-emergent adverse events | | | | | | |
| *Any AE* | 24 (63.2) | 22 (39.3) | | 30 (85.7); 304.6 | 23 (88.5); 359.0 | 24 (77.4); 277.7 |
| *Severe AE* | 6 (15.8) | 1 (1.8) | | 2 (5.7); 6.8 | 0 | 1 (3.2); 3.8 |
| *Serious AE* | 5 (13.2) | 1 (1.8) | | 3 (8.6); 10.2 | 1 (3.8); 4.4 | 2 (6.5); 7.5 |
| *AE possibly related to the study drug as assessed by the investigator* | 2 (5.3) | 13 (23.2) | | 13 (37.1); 84.6 | 12 (46.2); 110.8 | 7 (22.6); 56.3 |
| *AE leading to study drug discontinuation* | 2 (5.3) | 2 (3.6) | | 1 (2.9); 3.4 | 0 | 1 (3.2); 3.8 |
| *AE related to COVID-19* | 4 (10.5) | 0 | | 3 (8.6); 10.2 | 4 (15.4); 17.7 | 7 (22.6); 30.0 |
| *Death* | 0 | 0 | | 0 | 0 | 1 (3.2); 3.8^c^ |
| Treatment-emergent adverse events of special interest | | | | | | |
| *Adjudicated MACE* | 0 | 0 | | 0 | 0 | 0 |
| *Serious infections* | 1 (2.6) | 0 | | 1 (2.9); 3.4 | 0 | 0 |
| *Active tuberculosis* | 0 | 0 | | 0 | 0 | 0 |
| *Opportunistic infection^d^* | 0 | 0 | | 0 | 0 | 1 (3.2); 3.8 |
| *Herpes zoster* | 0 | 0 | | 0 | 0 | 0 |
| *Malignancies* | 0 | 0 | | 0 | 0 | 1 (3.2); 3.8 |
| *NMSC* | 0 | 0 | | 0 | 0 | 0 |
| *Hypersensitivity* | 1 (2.6) | 2 (3.6) | | 3 (8.6); 10.2 | 9 (34.6); 48.7 | 0 |
| *Serious hypersensitivity* | 0 | 0 | | 0 | 0 | 0 |
| *Adjudicated anaphylactic reactions* | 0 | 0 | | 0 | 0 | 0 |
| *Hepatic events* | 4 (10.5) | 2 (3.6) | | 1 (2.9); 10.2 | 0 | 1 (3.2); 3.8 |
| *Injection site reactions* | 0 | 1 (1.8) | | 0 | 2 (7.7); 8.9 | 2 (6.5); 7.5 |
| AE, adverse event; E/100 PY, events per 100 patient-years; JAK, Janus kinase; MACE, major adverse cardiovascular event; NMSC, nonmelanoma skin cancer; PBO, placebo; RZB, risankizumab.  ^a^Includes all patients who received ≥ 1 dose of the study drug in the induction study.  ^b^Includes all randomized patients who received IV RZB in the induction study and who also received ≥ 1 dose of study drug in the maintenance study.  ^c^Death was due to adenocarcinoma of the colon, considered unrelated to the study drug.  ^d^Excluding tuberculosis and herpes zoster. | | | | | | |

| **Table S4. Overview of Adverse Events for the Maintenance Study** | | | | | | |
| --- | --- | --- | --- | --- | --- | --- |
| **E, E/100 PY (%)** | **Non-AT-IR** | | | **AT-IR** | | |
|  | **PBO (Withdrawal) SC**  **N = 47**  **PY = 45.1** | **RZB 180 mg SC**  **N = 47**  **PY = 49.9** | **RZB 360 mg SC**  **N = 48**  **PY = 45.9** | **PBO (Withdrawal) SC**  **N = 149**  **PY = 129.8** | **RZB 180 mg SC**  **N = 146**  **PY = 135.5** | **RZB 360 mg SC**  **N = 147**  **PY = 127.6** |
| Overview of treatment-emergent adverse events | | | | | | |
| *Any AE* | 82 (182.0) | 87 (174.3) | 105 (228.6) | 317 (244.2) | 312 (230.3) | 301 (235.9) |
| *Severe AE* | 0 | 1 (2.0) | 0 | 14 (10.8) | 2 (1.5) | 7 (5.5) |
| *Serious AE* | 0 | 5 (10.0) | 0 | 20 (15.4) | 6 (4.4) | 11 (8.6) |
| *AE possibly related to the study drug as assessed by the investigator* | 10 (22.2) | 21 (42.1) | 15 (32.7) | 65 (50.1) | 64 (47.2) | 46 (36.1) |
| *AE leading to study drug discontinuation* | 0 | 0 | 0 | 4 (3.1) | 5 (3.7) | 5 (3.9) |
| *AE related to COVID-19* | 11 (24.4) | 8 (16.0) | 10 (21.8) | 17 (13.1) | 13 (9.6) | 19 (14.9) |
| *Death* | 0 | 0 | 0 | 0 | 0 | 1 (0.8)^a^ |
| Treatment-emergent adverse events of special interest | | | | | | |
| *Adjudicated MACE* | 0 | 0 | 0 | 0 | 0 | 0 |
| *Serious infections* | 0 | 1 (2.0) | 0 | 4 (3.1) | 1 (0.7) | 1 (0.8) |
| *Active tuberculosis* | 0 | 0 | 0 | 0 | 0 | 0 |
| *Opportunistic infection^b^* | 0 | 0 | 0 | 0 | 0 | 1 (0.8) |
| *Herpes zoster* | 0 | 1 (2.0) | 0 | 3 (2.3) | 1 (0.7) | 1 (0.8) |
| *Malignancies* | 0 | 0 | 0 | 1 (0.8) | 0 | 2 (1.6) |
| *NMSC* | 0 | 0 | 0 | 1 (0.8) | 0 | 0 |
| *Hypersensitivity* | 1 (2.2) | 1 (2.0) | 6 (13.1) | 9 (6.9) | 22 (16.2) | 9 (7.1) |
| *Serious hypersensitivity* | 0 | 0 | 0 | 0 | 0 | 0 |
| *Adjudicated anaphylactic reactions* | 0 | 0 | 0 | 0 | 0 | 0 |
| *Hepatic events* | 0 | 2 (4.0) | 13 (28.3) | 3 (2.3) | 1 (0.7) | 6 (4.7) |
| *Injection site reactions* | 0 | 6 (12.0) | 1 (2.2) | 3 (2.3) | 8 (5.9) | 9 (7.1) |
| AE, adverse event; AT-IR, with prior inadequate response or intolerance to advanced therapy; E/100 PY, events per 100 patient-years; MACE, major adverse cardiovascular event; NMSC, nonmelanoma skin cancer; Non-AT-IR, without prior inadequate response or intolerance to advanced therapy; PBO, placebo; RZB, risankizumab.  Includes all randomized patients who received IV RZB in the induction study and also received ≥ t 1 dose of study drug in the maintenance study.  ^a^Death was due to adenocarcinoma of the colon, considered unrelated to the study drug.  ^b^Excluding tuberculosis and herpes zoster. | | | | | | |
| **Table S5. Overview of Adverse Events Based on Number of Prior Advanced Therapies for the Maintenance Study** | | | | | | |
| **Patients, n (%); E/100 PY** | **AT-IR = 1** | | | **AT-IR ≥ 2** | | |
|  | **PBO (Withdrawal) SC**  **N = 64**  **PY = 58.8** | **RZB 180 mg SC**  **N = 56**  **PY = 54.4** | **RZB 360 mg SC**  **N = 60**  **PY = 55.0** | **PBO (Withdrawal) SC**  **N = 85**  **PY = 71.1** | **RZB 180 mg SC**  **N = 90**  **PY = 81.1** | **RZB 360 mg SC**  **N = 87**  **PY = 72.6** |
| Overview of treatment-emergent adverse events | | | | | | |
| *Any AE* | 49 (76.6); 207.6 | 43 (76.8); 187.5 | 36 (60.0); 141.8 | 65 (76.5); 274.4 | 66 (73.3); 259.0 | 66 (75.9); 307.3 |
| *Severe AE* | 5 (7.8); 10.2 | 2 (3.6); 3.7 | 1 (1.7); 1.8 | 5 (5.9); 11.3 | 0 | 5 (5.7); 8.3 |
| *Serious AE* | 6 (9.4); 11.9 | 3 (5.4); 5.5 | 2 (3.3); 3.6 | 10 (11.8); 18.3 | 3 (3.3); 3.7 | 8 (9.2); 12.4 |
| *AE possibly related to the study drug as assessed by the investigator* | 11 (17.2); 20.4 | 6 (10.7); 23.9 | 8 (13.3); 20.0 | 24 (28.2); 74.6 | 24 (26.7); 62.9 | 17 (19.5); 48.2 |
| *AE leading to study drug discontinuation* | 2 (3.1); 5.1 | 0 | 1 (1.7); 1.8 | 1 (1.2); 1.4 | 3 (3.3); 6.2 | 4 (4.6); 5.5 |
| *AE related to COVID-19* | 9 (14.1); 15.3 | 2 (3.6); 3.7 | 3 (5.0); 5.5 | 8 (9.4); 11.3 | 11 (12.2); 13.6 | 13 (14.9); 22.0 |
| *Death* | 0 | 0 | 0 | 0 | 0 | 1 (1.1); 1.4^a^ |
| Treatment-emergent adverse events of special interest | | | | | | |
| *Adjudicated MACE* | 0 | 0 | 0 | 0 | 0 | 0 |
| *Serious infections* | 1 (1.6); 1.7 | 1 (1.8); 1.8 | 0 | 3 (3.5); 4.2 | 0 | 1 (1.1); 1.4 |
| *Active tuberculosis* | 0 | 0 | 0 | 0 | 0 | 0 |
| *Opportunistic infection^b^* | 0 | 0 | 0 | 0 | 0 | 1 (1.1); 1.4 |
| *Herpes zoster* | 2 (3.1); 3.4 | 1 (1.8); 1.8 | 0 | 1 (1.2); 1.4 | 0 | 1 (1.1); 1.4 |
| *Malignancies* | 0 | 0 | 0 | 1 (1.2); 1.4 | 0 | 2 (2.3); 2.8 |
| *NMSC* | 0 | 0 | 0 | 1 (1.2); 1.4 | 0 | 0 |
| *Hypersensitivity* | 6 (9.4); 10.2 | 4 (7.1); 7.4 | 2 (3.3); 7.3 | 3 (3.5); 4.2 | 15 (16.7); 22.2 | 3 (3.4); 6.9 |
| *Serious hypersensitivity* | 0 | 0 | 0 | 0 | 0 | 0 |
| *Adjudicated anaphylactic reactions* | 0 | 0 | 0 | 0 | 0 | 0 |
| *Hepatic events* | 0 | 1 (1.8); 1.8 | 3 (5.0); 5.5 | 1 (1.2); 4.2 | 0 | 3 (3.4); 4.1 |
| *Injection site reactions* | 1 (1.6); 1.7 | 1 (1.8); 3.7 | 0 | 1 (1.2); 2.8 | 5 (5.6); 7.4 | 4 (4.6); 12.4 |
| AE, adverse event; AT-IR, with prior inadequate response or intolerance to advanced therapy; E/100 PY, events per 100 patient-years; MACE, major adverse cardiovascular event; NMSC, nonmelanoma skin cancer; PBO, placebo; RZB, risankizumab.  Includes all randomized patients who received IV RZB in the induction study and also received ≥ 1 dose of study drug in the maintenance study.  ^a^Death was due to adenocarcinoma of the colon, considered unrelated to the study drug.  ^b^Excluding tuberculosis and herpes zoster. | | | | | | |

**Figure S1. AT-IR Categorization and Patient Disposition in the Induction and Maintenance Studies**


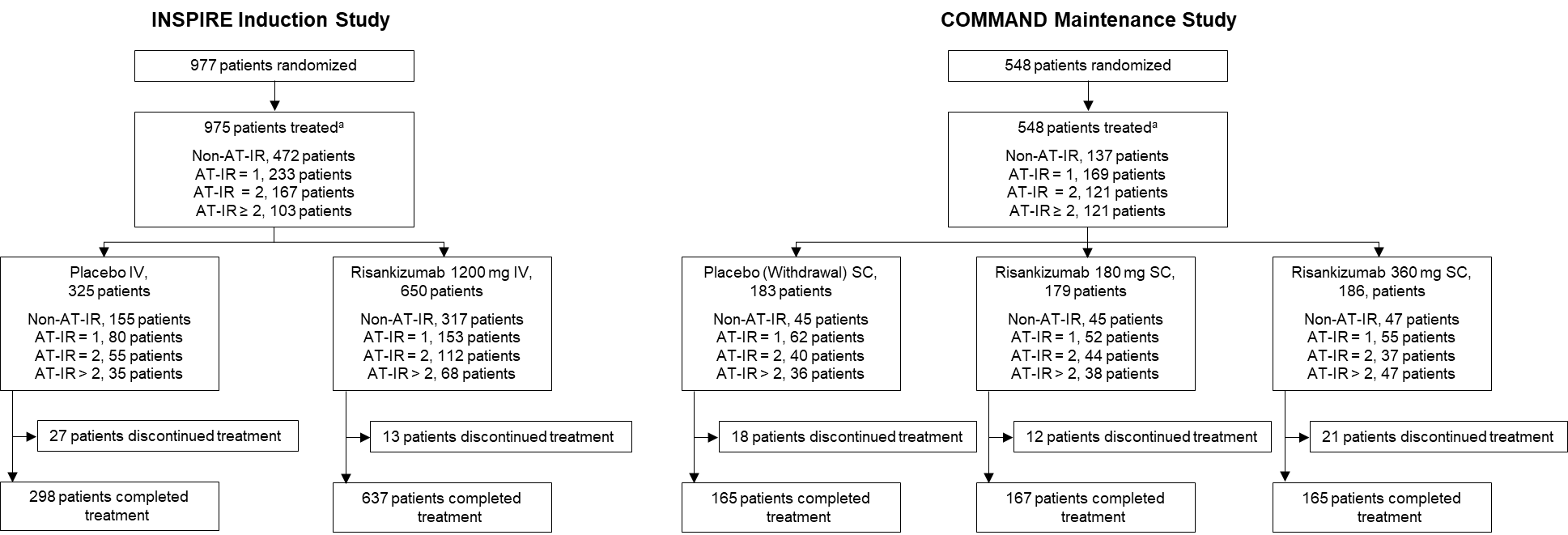


AT-IR, prior inadequate response or intolerance to advanced therapy; IV, intravenous; Non-AT-IR, without prior inadequate response or intolerance to advanced therapy; SC, subcutaneous.
^a^Represents the patient population included in the efficacy analyses.

**Figure S2. Week 12 Efficacy Outcomes by Prior TNF Inhibitor to Which Patients Had Prior Inadequate Response or Intolerance**


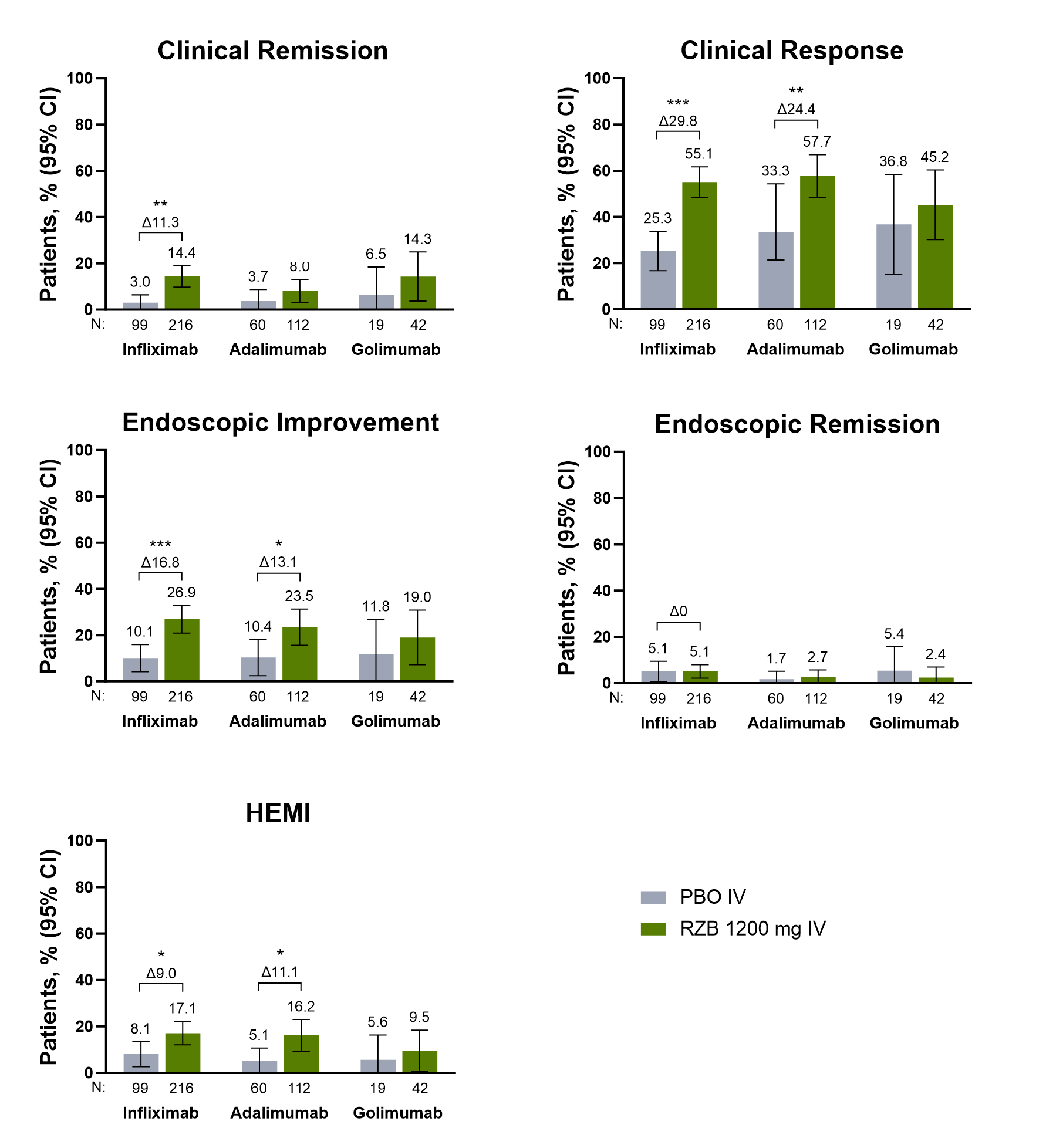


CI, confidence interval; HEMI, histologic endoscopic mucosal improvement; IV, intravenous; PBO, placebo; RZB, risankizumab; TNF, tumor necrosis factor.
Nominal **P* ≤ .05, ***P* ≤ .01, and ****P* ≤ .001.

**Figure S3. Week 52 Efficacy Outcomes by Prior TNF Inhibitor to Which Patients Had Prior Inadequate Response or Intolerance**


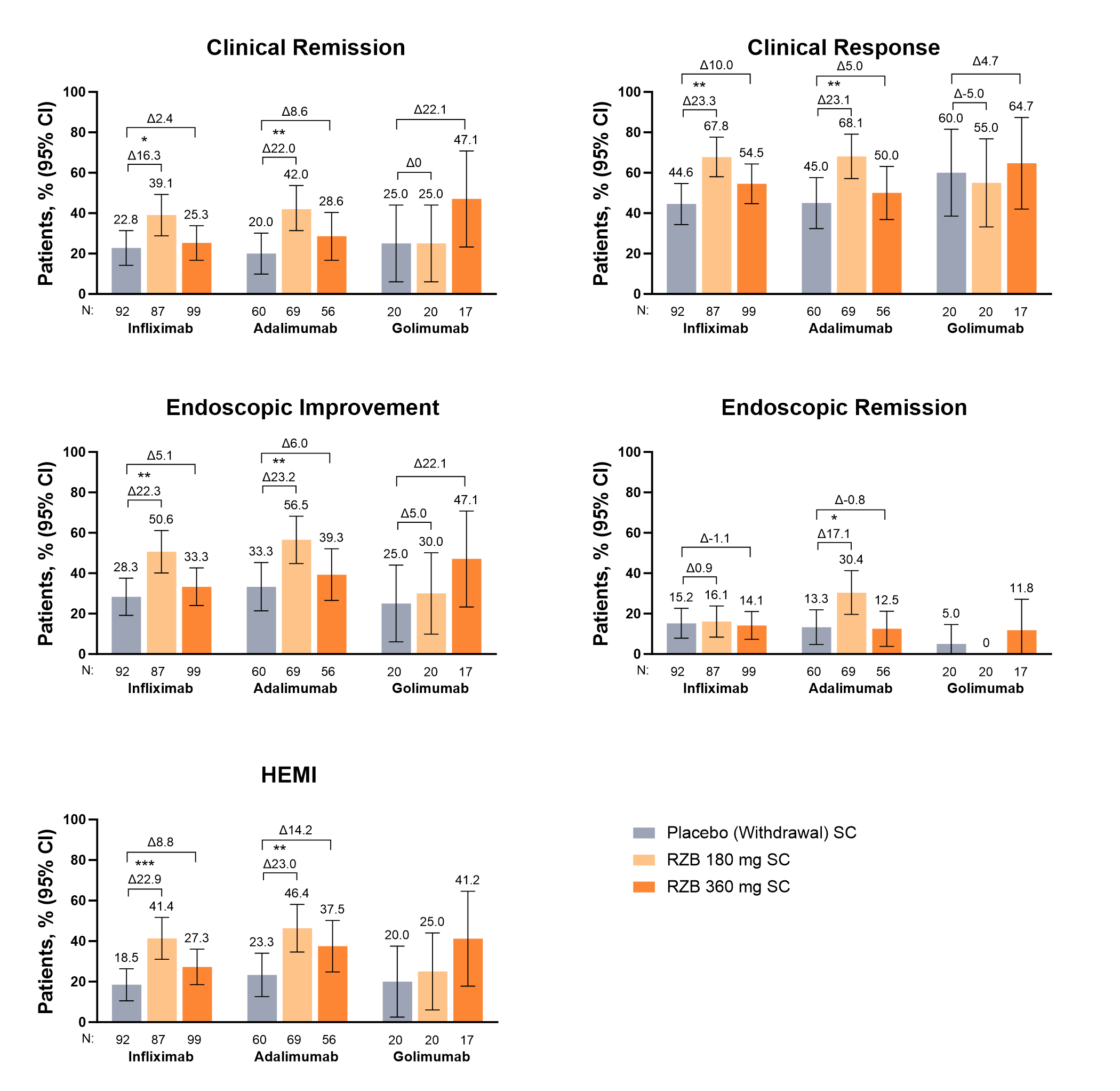
CI, confidence interval; HEMI, histologic endoscopic mucosal improvement; PBO, placebo; RZB, risankizumab; SC, subcutaneous; TNF, tumor necrosis factor.
Nominal **P* ≤ .05, ***P* ≤ .01, and ****P* ≤ .001.

**Figure S4. Week 12 and Week 52 Clinical Remission per Adapted Mayo Score by Prior Failure to Advanced Therapy**

**
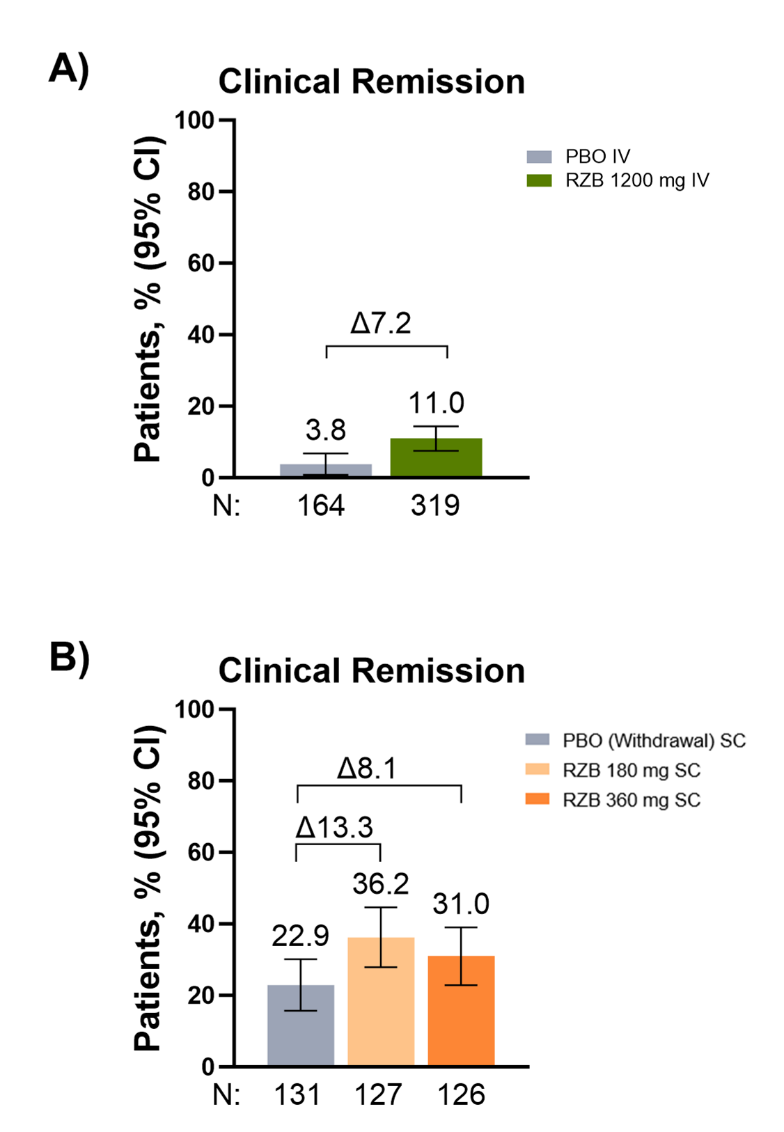
**

CI, confidence interval; IV, intravenous; PBO, placebo; RZB, risankizumab; SC, subcutaneous.

Includes patients who at least have failed an advanced therapy due to lack of efficacy. Patients with intolerance to advanced therapy were removed from this analysis.

| **Table S6. Overview of Adverse Events for the Induction and Maintenance Studies** **by Prior Failure to Advanced Therapy** | | | | | | | |
| --- | --- | --- | --- | --- | --- | --- | --- |
|  | **Week 12 of Induction** | |  | **Week 52 of Maintenance** | | |  |
| **Patients, n (%)** | **PBO**  **IV**  **N = 164** | **RZB 1200 mg IV**  **N = 319** |  | **PBO (Withdrawal) SC**  **N = 142** | **RZB 180 mg SC**  **N = 139** | **RZB 360 mg SC**  **N = 133** |  |
| Overview of treatment-emergent adverse events | | | | | | |  |
| *Any AE* | 86 (52.4) | 140 (43.9) |  | 107 (75.4) | 105 (75.5) | 94 (70.7) |  |
| *Severe AE* | 20 (12.2) | 4 (1.3) |  | 10 (7.0) | 2 (1.4) | 5 (3.8) |  |
| *Serious AE* | 20 (12.2) | 5 (1.6) |  | 15 (10.6) | 6 (4.3) | 9 (6.8) |  |
| *AE possibly related to the study drug as assessed by the investigator* | 14 (8.5) | 40 (12.5) |  | 33 (23.2) | 30 (21.6) | 24 (18.0) |  |
| *AE leading to study drug discontinuation* | 7 (4.3) | 3 (0.9) |  | 2 (1.4) | 3 (2.2) | 4 (3.0) |  |
| *AE related to COVID-19* | 11 (6.7) | 23 (7.2) |  | 17 (12.0) | 13 (9.4) | 15 (11.3) |  |
| *Deaths* | 0 | 1 (0.3) |  | 0 | 0 | 1 (0.8) |  |
| Treatment-emergent adverse events of special interest | | | | | | |  |
| *Adjudicated MACE* | 0 | 0 |  | 0 | 0 | 0 |  |
| *Serious infections* | 2 (1.2) | 2 (0.6) |  | 4 (2.8) | 1 (0.7) | 1 (0.8) |  |
| *Active tuberculosis* | 0 | 0 |  | 0 | 0 | 0 |  |
| *Opportunistic infection^a^* | 0 | 0 |  | 0 | 0 | 1 (0.8) |  |
| *Herpes zoster* | 0 | 1 (0.3) |  | 3 (2.1) | 1 (0.7) | 1 (0.8) |  |
| *Malignancies* | 2 (1.2) | 0 |  | 1 (0.7) | 0 | 1 (0.8) |  |
| *NMSC* | 0 | 0 |  | 1 (0.7) | 0 | 0 |  |
| *Hypersensitivity* | 3 (1.8) | 16 (5.0) |  | 9 (6.3) | 18 (12.9) | 5 (3.8) |  |
| *Serious hypersensitivity* | 0 | 0 |  | 0 | 0 | 0 |  |
| *Adjudicated anaphylactic reactions* | 0 | 0 |  | 0 | 0 | 0 |  |
| *Hepatic events* | 10 (6.1) | 6 (1.9) |  | 1 (0.7) | 1 (0.7) | 6 (4.5) |  |
| *Injection site reactions* | 2 (1.2) | 2 (0.6) |  | 2 (1.4) | 6 (4.3) | 4 (3.0) |  |

AE, adverse event; MACE, major adverse cardiovascular event; NMSC, nonmelanoma skin cancer; PBO, placebo; RZB, risankizumab.

Includes all randomized patients who received IV RZB in the induction study and also received ≥ 1 dose of study drug in the maintenance study.

Includes patients who at least have failed an advanced therapy due to lack of efficacy. Patients with intolerance to advanced therapy were removed from this analysis.

^a^Excluding tuberculosis and herpes zoster.
